# Microsatellite genotyping and genome-wide single nucleotide polymorphism-based indices of *Plasmodium falciparum* diversity within clinical infections

**DOI:** 10.1186/s12936-016-1324-4

**Published:** 2016-05-12

**Authors:** Lee Murray, Victor A. Mobegi, Craig W. Duffy, Samuel A. Assefa, Dominic P. Kwiatkowski, Eugene Laman, Kovana M. Loua, David J. Conway

**Affiliations:** Pathogen Molecular Biology Department, London School of Hygiene and Tropical Medicine, Keppel St, London, WC1E 7HT UK; Medical Research Council Unit, Fajara, Atlantic Road, Banjul, Gambia; Wellcome Trust Sanger Institute, Hinxton, Cambridgeshire, UK; National Institute of Public Health, Conakry, Republic of Guinea

## Abstract

**Background:**

In regions where malaria is endemic, individuals are often infected with multiple distinct parasite genotypes, a situation that may impact on evolution of parasite virulence and drug resistance. Most approaches to studying genotypic diversity have involved analysis of a modest number of polymorphic loci, although whole genome sequencing enables a broader characterisation of samples.

**Methods:**

PCR-based microsatellite typing of a panel of ten loci was performed on *Plasmodium falciparum* in 95 clinical isolates from a highly endemic area in the Republic of Guinea, to characterize within-isolate genetic diversity. Separately, single nucleotide polymorphism (SNP) data from genome-wide short-read sequences of the same samples were used to derive within-isolate fixation indices (*F*_ws_), an inverse measure of diversity within each isolate compared to overall local genetic diversity. The latter indices were compared with the microsatellite results, and also with indices derived by randomly sampling modest numbers of SNPs.

**Results:**

As expected, the number of microsatellite loci with more than one allele in each isolate was highly significantly inversely correlated with the genome-wide *F*_ws_ fixation index (r = −0.88, P < 0.001). However, the microsatellite analysis revealed that most isolates contained mixed genotypes, even those that had no detectable genome sequence heterogeneity. Random sampling of different numbers of SNPs showed that an *F*_ws_ index derived from ten or more SNPs with minor allele frequencies of >10 % had high correlation (r > 0.90) with the index derived using all SNPs.

**Conclusions:**

Different types of data give highly correlated indices of within-infection diversity, although PCR-based analysis detects low-level minority genotypes not apparent in bulk sequence analysis. When whole-genome data are not obtainable, quantitative assay of ten or more SNPs can yield a reasonably accurate estimate of the within-infection fixation index (*F*_ws_).

**Electronic supplementary material:**

The online version of this article (doi:10.1186/s12936-016-1324-4) contains supplementary material, which is available to authorized users.

## Background

Malaria parasite blood-stage infections commonly contain a mixture of different haploid parasite genotypes, particularly in areas of high endemicity where superinfection frequently occurs [1]. Cross-mating and recombination between different genomes of parasites occurring together in a vector mosquito blood meal is, therefore, most frequent in highly endemic areas, whereas in areas of low endemicity inbreeding may be common as most infections contain single or highly related genotypes [1–6]. In an experimental model of malaria using *Plasmodium chabaudi* in mice, multiple genotype infections have been associated with apparent short-term evolution of virulence [7], alterations to parasite sex ratio and production of gametocytes [8, 9], and effects on the immune clearance rate [10]. If such processes occur in human malaria, they might also impact on drug resistance evolution [3, 11].

Previous analyses of within-host diversity of *P. falciparum*, the causative agent of most human malaria cases globally [12], have typically involved genotyping a small number of highly polymorphic gene loci [13], or multiple putatively neutral microsatellite marker loci [1]. These have demonstrated wide variation in the genotypic complexity of infections among geographical populations of *P. falciparum*, which inversely correlates with local levels of multilocus linkage disequilbrium [1, 14–17]. Further dissection of the within-host diversity of *P. falciparum* infections has been performed using genome-wide single nucleotide polymorphism (SNP) data, showing that a high degree of relatedness is seen among some distinct parasite clones within infections, in comparison to those sampled from separate infections [4, 18]. This illustrates that a multiple genotype *P. falciparum* infection may be comprised of a mixture of closely related, non-identical parasites, or multiple genetically unrelated parasites, or it may be a complex mixture of both.

The relative proportions of SNP alleles in whole-genome sequence data from an infection can be estimated from the proportions of reads mapping to a reference sequence, and this allows computation of a within-isolate fixation index, *F*_ws_ [19, 20]. This index compares within-host diversity (‘w’) to that which exists in the overall local parasite population (or sub-population, ‘s’). It has a possible range from zero (when the sample from an infection contains all possible diversity) through to 1.0 (when the sample from an infection contains no sequence diversity), and this is profoundly influenced by the relative proportions of genotypes in the case of a mixed infection.

Here, the within-host diversity of *P. falciparum* within a highly endemic population in West Africa has been characterized using two distinct methods. Firstly, microsatellite PCR-based genotyping was performed with a panel of ten loci widely distributed in the parasite genome, and then whole-genome sequence data from the same samples were used to analyse SNPs in order to compute the *F*_ws_ indices. The relationship between these different types of estimates is examined, and the use of small numbers of SNPs to derive *F*_ws_ indices is also illustrated, so that the potential value of SNP genotyping may be considered when whole-genome data are not obtainable.

## Methods

### *Plasmodium falciparum* sample collection and preparation

Patients between the ages of one and 9 years presenting with uncomplicated *P. falciparum* malaria, as confirmed by rapid diagnostic test (Paracheck, Orchid Biomedical systems, India), were recruited from local health facilities within a 25-km radius of the town of N’Zerekore in the Republic of Guinea, between March and May 2011. Written informed consent was obtained from a parent or guardian of each child sampled, and all patients diagnosed with malaria were treated with artesunate-amodiaquine regardless of study participation. Ethical approval for the sample collection for parasite sequencing and genotypic analysis was obtained from the *Comité d’Ethique National pour la Recherche en Santé, République de Guinée* (National Ethics Committee for Health Research, Republic of Guinea). Following detection of *P. falciparum* infection, up to 5 mL of venous blood was collected in EDTA (ethylenediaminetetraacetic acid) vacutainers from consenting subjects and leukocyte depletion of each sample was performed using CF11 cellulose column filtration [21], and the erythrocytes were then frozen at −20 °C prior to shipment on dry ice to The Gambia. DNA extraction was carried out at the MRC Laboratories in The Gambia, using a QIAamp Blood Midi Kit (Qiagen) and DNA was quantified using a NanoDrop ND-1000 v3.3 spectrophotometer (NanoDrop Technologies, USA). Separate aliquots of DNA from each individual sample were then used for microsatellite genotyping and whole genome sequencing. The illumina paired-end, short-read genome sequencing of these samples has been previously described [22].

### Microsatellite genotyping and analysis of *Plasmodium falciparum* clinical isolates

Microsatellite genotyping was performed on 95 isolates, out of the 100 for which genome sequence data were separately published [22]. Ten polymorphic microsatellite loci were genotyped using previously described heminested PCR methods [23], with dye-labelled internal primers as specified for each locus in a recent analysis of other samples from West Africa [16]. The PCR products were run electrophoretically on an ABI 3130XL Genetic analyzer alongside a GeneScan™ 500 LIZ internal size control standard. For each of the microsatellite loci for which multiple product sizes were detected within an isolate, the majority and minor alleles for each isolate were recorded [16]. The number of different genotypes detectable within an isolate was counted as the maximum number of alleles found at any individual microsatellite locus. The proportion of loci that had more than one allele detectable within each isolate was also counted as a separate measure of mixedness. For analyses of population-wide frequencies the majority allele at each microsatellite locus within each isolate was counted.

### Genome-wide calculation of the within-isolate fixation index *F*_*ws*_

The whole genome illumina paired-end, short-read sequence data from the *P. falciparum* clinical isolates genotyped here are published and available at the European Nucleotide Archive as previously described [22]. Quantification of within-host parasite diversity within each individual isolate relative to the overall local population diversity was performed using the *F*_ws_ metric [19, 20]. For this process, using a set of 50,082 biallelic SNPs, allele frequencies were calculated at every SNP position for each isolate individually, with *p* and *q* representing the proportions of read counts for the minor and major alleles. All SNPs were then assigned to ten minor allele frequency (MAF) intervals representing the proportional frequency of the minor allele at each SNP across the Guinean population, with the ten equally sized intervals ranging from 0–5 % up to 45–50 %. Levels of within-host (*H*_w_) and local parasite sub-population (*H*_s_) heterozygosity for each SNP were calculated as *H*_w_ = 2*p_w_*q_w_ and *H*_s_ = 2*p_s_*q_s_. The mean *H*_w_ and *H*_s_ of each MAF interval were then computed from the corresponding heterozygosity scores of all SNPs within that particular interval. The resulting—plot of *H*_*w*_ against *H*_*s*_ for each isolate was produced and a linear regression model was used to determine a value for the gradient *H*_w_/*H*_s−_, with *F*_ws_ = 1−(*H*_w_/*H*_s_). All *F*_ws_ analyses were performed using custom scripts in R.

### Estimation of the *F*_*ws*_ index from limited numbers of SNPs

*F*_*ws*_ indices were then derived from sampling a small number (between one and 20) of randomly selected SNPs and compared with the genome-wide indices previously determined. A Pearson’s correlation coefficient with the genome-wide SNP estimate of *F*_ws_ was determined across all isolates. For each limited SNP selection of *n* SNPs, 100 random samples of *n* SNPs were analysed and the mean Pearson’s coefficient observed for these was calculated. This analysis was repeated and comparisons performed between sets of SNPs with different ranges of overall minor allele frequencies.

## Results

### Mixed genotype infections assessed by multi-locus microsatellite analysis

**Fig. 1:**
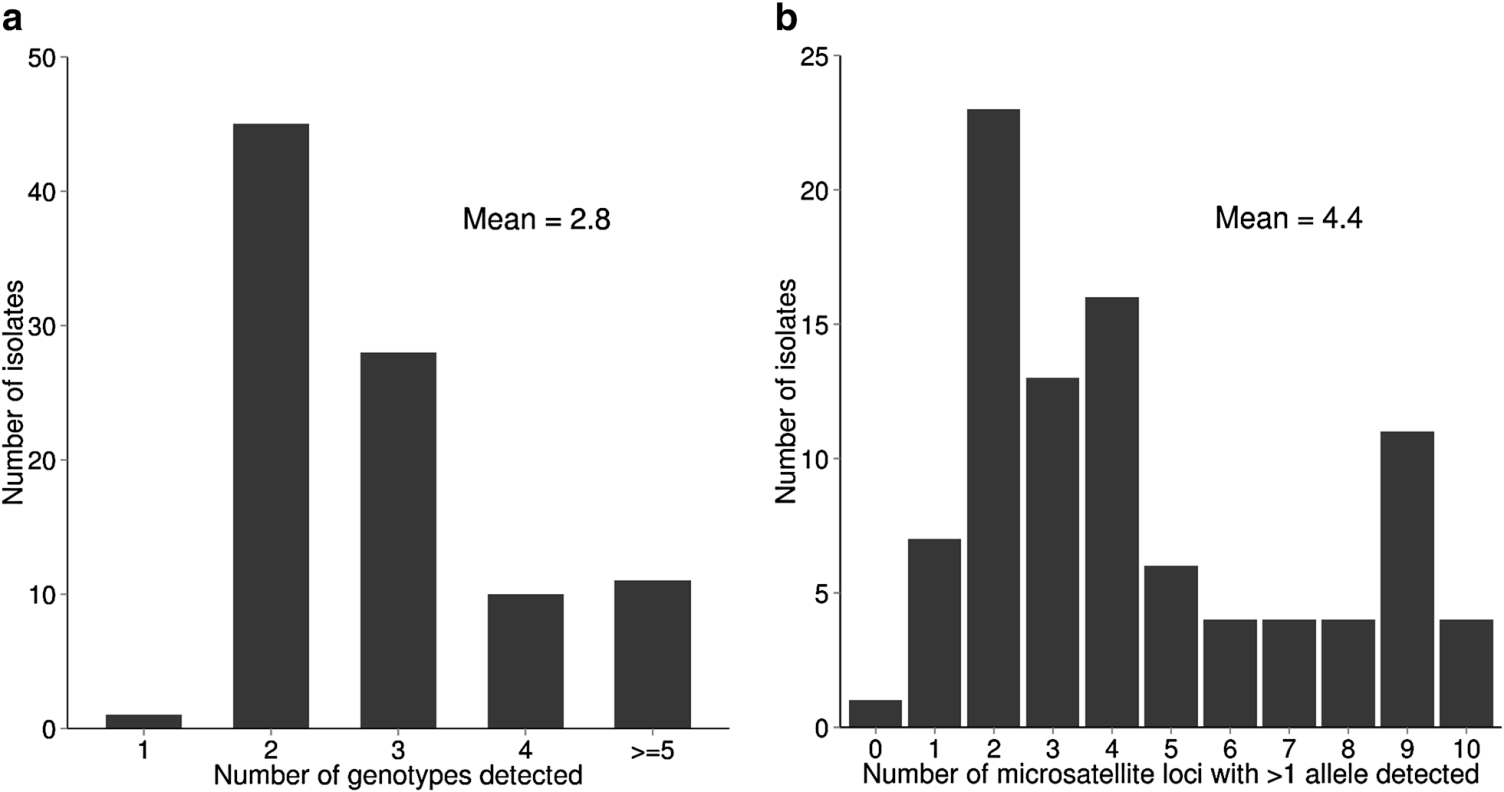
Multiple *Plasmodium falciparum* genotypes in blood samples from patients with clinical malaria in Guinea assessed by genotyping with ten microsatellite loci. **a** Numbers of clinical isolates with given numbers of different *P. falciparum* genotypes detected (total N = 95). **b** Numbers of clinical isolates with given numbers of *P. falciparum* loci having >1 detectable allele out of ten loci genotyped (total N = 93 with complete ten-locus genotype profiles)

A ten-locus microsatellite analysis was used to generate genotype profiles for each of the 95 Guinean *P. falciparum* clinical isolates (Additional file 1). A mean of 2.8 different genotypes was detected per isolate. Multiple genotypes were detected in all except one of the isolates, with 45 isolates (47.4 %) having two genotypes, 28 (29.4 %) having three, ten (10.5 %) having four, and 11 isolates (11.5 %) having five or more genotypes (Fig. 1a). Within each isolate, the numbers of loci that had multiple alleles detected had a wide range, with a mean of 4.4 out of the ten loci (Fig. 1b). The majority allele at each locus within each of the isolates was counted to assess overall population frequencies, which were similar to those seen in a sample of isolates taken from this area in the previous year [16] (Additional file 2).

### Genotypic mixedness estimated from whole-genome SNP data compared with microsatellite data

Genome-wide SNP data for each of the isolates were used to generate within-host *F*_ws_ fixation indices, for comparison to the microsatellite profiling of the same isolates. Using the illumina short-read sequence reads to estimate relative frequencies of SNP alleles within each isolate, the within-isolate diversity (*H*_w_) was derived, and the gradient of *H*_w_/*H*_s_ (where *H*_s_ is the diversity in the entire local population sample) was calculated to derive the *F*_ws_ index for each isolate (with a range from zero indicating maximal possible diversity, to 1.0 indicating no observed diversity within an isolate). Over all 95 clinical isolates, the mean genome-wide *F*_ws_ index was 0.79, with a range of 0.16 to 1.00 (Additional file 1). Of these, 50 isolates (52.6 %) had an *F*_ws_ index approaching 1 (values of >0.95) indicating that they each contain a single predominant haploid genome sequence, with any additional genotypes being rare or absent, or closely related to the predominant genotype. Conversely, 45 of the clinical isolates (47.4 %) had an *F*_ws_ index of <0.95 and were thus clearly genotypically mixed infections. No significant correlation was seen between average genome sequence read mapping depth and *F*_ws_ index (Pearson’s r = 0.02), indicating that there was no bias in estimating within-host diversity due to sequence coverage.

**Fig. 2:**
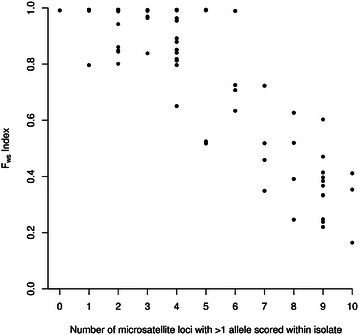
Concordance between microsatellite genotyping and *F*_ws_ within-infection fixation indices, derived from genome-wide SNP data for *Plasmodium falciparum* clinical isolates from Guinea (N = 93 with complete ten-locus microsatellite genotype profiles). As expected, a highly negative relationship (Pearson’s coefficient = −0.88, P < 0.001) was seen between the *F*_ws_ fixation index and the number of microsatellite loci with >1 allele detected within each infection. The numbers of clinical isolates within each x-axis category are shown in Fig. 1b

Microsatellite typing allows an assessment of the proportion of loci that have multiple alleles within an infection, as well as a minimum estimate of the number of different parasite genotypes (the highest number of alleles detected at any locus). As expected, there was a highly significant negative correlation between the *F*_ws_ fixation index derived from the SNP analysis and the number of microsatellite loci with multiple alleles detected within each infection (Pearson’s r = −0.88, P < 0.001) (Fig. 2). There was also a highly significant negative correlation between the isolate *F*_ws_ index and the number of different genotypes within each infection detected by microsatellite typing (Pearson’s r = −0.80, P < 0.001). However, many infections that appeared to be unmixed on the basis of the genome sequence data (having an *F*_ws_ index of >0.95) were shown to have multiple genotypes as assessed by PCR-based microsatellite genotyping, with some of these infections having multiple alleles at several of the microsatellite loci (Fig. 2; Additional file 1).

### Identifying SNPs for assay of within-host diversity with a small number of loci

**Fig. 3:**
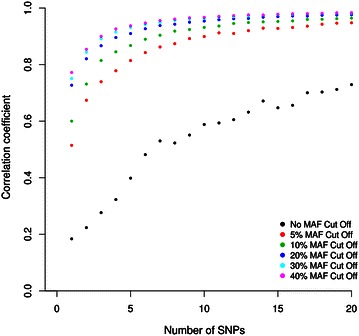
Correlation coefficients of *Plasmodium falciparum* *F*_ws_ within-infection fixation indices derived from randomly sampled small numbers of SNPs, compared with indices using all genome-wide SNPs analysed. The effect of using SNPs with allele frequencies above different levels is shown. Within the overall SNP set sampled, 7684 SNPs have a minor allele frequency (MAF) of >5 %; 5004 SNPs have MAF of >10 %; 2854 SNPs have MAF of >20 %; 1719 SNPs have MAF >30 %; 833 SNPs have MAF >40 %. Replications of 100 random samples of different numbers of SNPs (between 1 and 20) were performed, and the mean correlation coefficient for each is plotted

Calculation of *F*_ws_ indices as above was performed with genome-wide SNP data for each infection, although such data will be rarely available for new clinical samples. In order to investigate the potential of using small numbers of SNPs, data from up to 20 randomly chosen SNPs were used to estimate *F*_ws_ indices, and correlations with values derived from genome-wide SNP data were determined (Fig. 3). By selecting from all SNPs, regardless of allele frequency, correlations with the genome-wide estimate were modest (for 20 SNPs Pearson’s r < 0.71). However, by selecting SNPs with minor allele frequencies greater than a specified threshold (either <5, 10, 20, 30, or 40 %), much higher correlations were seen. Correlation for each selection began to plateau above ~ten SNPs (Fig. 3). For example, randomly choosing ten or more SNPs with minor allele frequencies of greater than 10 % gave correlations of r > 0.9 in comparisons with the genome-wide *F*_ws_ indices.

## Discussion

A combination of microsatellite locus typing and genome-wide estimation of SNP-based allele frequencies has been used here to characterize *P. falciparum* diversity within clinical infections. The results are indicative of a high degree of transmission intensity in the Guinean population studied, and are consistent with previous microsatellite data from other samples taken locally [16]. Interestingly, the genome-wide SNP data here indicate more than half of all infections to each be composed predominantly of single genotypes, whereas microsatellite genotyping detected additional genotypes within infections. Microsatellite typing allows the sensitive detection of distinct parasite genotypes present at low proportions within an infection, although cloning or single-cell analysis of isolates would be needed to estimate the degree of relatedness among the different parasites [4, 18, 24].

Random sampling of the genome-wide SNP data shows that the within-isolate *F*_ws_ fixation indices may be estimated from modest numbers of SNPs, and correlated with the indices derived from genome-wide data. Therefore, to estimate genotypic mixedness of isolates without whole genome sequencing, it may be feasible to quantitate alleles of between ten and 20 SNPs with other genotyping tools, particularly focusing on SNPs with high overall minor allele frequencies. It is preferable that these SNPs should be neutral, so that estimates are not biased by selection acting on the parasite.

Understanding processes affecting different parasite genotypes within an infection could offer insight into mechanisms that are clinically relevant. Genome sequencing allows broad or deep sampling of diversity within infections [20, 25], but resolution of individual parasite clone genotypes is currently achievable only through either extensive limiting dilution cloning [4] or single cell genome analysis [18]. Previous studies have shown that proportions of clones in peripheral blood of infected humans varies over time, and can show marked differences between successive days [26]. It is possible that some clones within an infection exist at low proportions due to competitive suppression by other *P. falciparum* genotypes, or specific selection by the host due to immunity or receptor polymorphisms. Further dissection of the patterns of parasite genotypic diversity in clinical isolates, and possible interactions between genotypes, may lead to novel understanding of malaria parasites which will be relevant for disease control and potential future elimination [6, 27].

## Conclusions

This study shows that estimates of genotypic complexity of malaria parasite infections using very different methods give correlated and complementary information. The within-infection fixation index *F*_ws_ yields a standardized inverse measure (within-infection diversity being 1 − *F*_ws_) which may be derived from genome-wide short read sequence data if this is available, or alternatively can be estimated from a modest number of randomly sampled SNPs which could be genotyped by other methods. Multilocus microsatellite PCR-based genotyping gives estimates of infection complexity that correlate strongly with those from the SNP analyses, while also being more sensitive to detect additional genotypes in some infections that appear to have unmixed sequences. With a wide range of methods now available, studies can choose genotyping and analytical approaches to suit investigational goals, recognizing relative advantages of each in relation to the costs and available resources.

## Additional files

10.1186/s12936-016-1324-4 Microsatellite genotype profiles of 95 Guinean *P. falciparum* clinical isolates as ranked by isolate *F*_ws_ scores.
10.1186/s12936-016-1324-4 Allele frequencies at 10 microsatellite loci in population samples of *Plasmodium falciparum* clinical isolates from N’Zerekore in the Republic of Guinea.
