# Supplementary material for: Microsatellite genotyping and genome-wide single nucleotide polymorphism-based indices of Plasmodium falciparum diversity within clinical infections
Source: Malar J. 2016 May 12;15:275. doi: 10.1186/s12936-016-1324-4 (PMC4865991; doi:10.1186/s12936-016-1324-4)
Supplement: Supplementary file 2 — 10.1186/s12936-016-1324-4 Allele frequencies at 10 microsatellite loci in population samples of Plasmodium falciparum clinical isolates from N’Zerekore in the Republic of Guinea. [file 12936_2016_1324_MOESM2_ESM.docx]

**Supplementary Table S2.** Allele frequencies at 10 microsatellite loci in population samples of *Plasmodium falciparum* clinical isolates from N’Zerekore in the Republic of Guinea.

| Locus | Allele | | Current Study | Previous Survey * |
| --- | --- | --- | --- | --- |
| *TA1* | 145 | 0.011 | | 0 |
|  | 151 | 0.011 | | 0 |
|  | 157 | 0.032 | | 0 |
|  | 160 | 0.179 | | 0.093 |
|  | 163 | 0.063 | | 0.256 |
|  | 166 | 0.200 | | 0.256 |
|  | 169 | 0.179 | | 0.093 |
|  | 172 | 0.126 | | 0.093 |
|  | 175 | 0.053 | | 0.093 |
|  | 178 | 0.074 | | 0.023 |
|  | 181 | 0.032 | | 0.093 |
|  | 184 | 0.032 | | 0 |
|  | 193 | 0.011 | | 0 |
|  | n | 95 | | 43 |
|  |  |  | |  |
| *TA87* | 90 | 0.021 | | 0 |
|  | 93 | 0.074 | | 0 |
|  | 96 | 0.021 | | 0.045 |
|  | 99 | 0.116 | | 0.205 |
|  | 102 | 0.200 | | 0.227 |
|  | 105 | 0.200 | | 0.227 |
|  | 108 | 0.168 | | 0.114 |
|  | 111 | 0.137 | | 0.091 |
|  | 114 | 0.021 | | 0.023 |
|  | 117 | 0.021 | | 0.045 |
|  | 120 | 0.03 | | 0.023 |
|  | 123 | 0.021 | | 0 |
|  | n | 95 | | 44 |
|  |  |  | |  |
| *ARA2* | 50 | 0 | | 0 |
|  | 56 | 0 | | 0.045 |
|  | 59 | 0.179 | | 0.045 |
|  | 62 | 0.095 | | 0.114 |
|  | 65 | 0.232 | | 0.227 |
|  | 68 | 0.189 | | 0.136 |
|  | 71 | 0.179 | | 0.068 |
|  | 74 | 0.095 | | 0.091 |
|  | 77 | 0 | | 0.045 |
|  | 80 | 0.011 | | 0.023 |
|  | 83 | 0.011 | | 0 |
|  | 86 | 0 | | 0 |
|  | 89 | 0 | | 0 |
|  | 92 | 0.011 | | 0 |
|  | 95 | 0 | | 0.023 |
|  | 98 | 0 | | 0.182 |
|  | n | 95 | | 44 |
|  |  |  | |  |
| *Pfg377* | 85 | 0 | | 0.119 |
|  | 91 | 0.021 | | 0.024 |
|  | 94 | 0.053 | | 0.095 |
|  | 97 | 0.242 | | 0.167 |
|  | 100 | 0.579 | | 0.571 |
|  | 103 | 0.074 | | 0.024 |
|  | 106 | 0.021 | | 0 |
|  | 118 | 0.011 | | 0 |
|  | n | 95 | | 42 |
|  |  |  | |  |
| *PfPK2* | 139 | 0.011 | | 0 |
|  | 154 | 0 | | 0.023 |
|  | 157 | 0.011 | | 0.045 |
|  | 160 | 0.126 | | 0.205 |
|  | 163 | 0.200 | | 0.182 |
|  | 166 | 0.189 | | 0.205 |
|  | 169 | 0.168 | | 0.091 |
|  | 172 | 0.084 | | 0.068 |
|  | 175 | 0.095 | | 0.136 |
|  | 178 | 0.063 | | 0.023 |
|  | 181 | 0.032 | | 0.023 |
|  | 184 | 0 | | 0 |
|  | 187 | 0.011 | | 0 |
|  | 190 | 0.011 | | 0 |
|  | n | 95 | | 44 |
|  |  |  | |  |
| *POLYα* | 117 | 0 | | 0 |
|  | 120 | 0 | | 0 |
|  | 123 | 0 | | 0.023 |
|  | 129 | 0 | | 0.023 |
|  | 132 | 0.011 | | 0 |
|  | 135 | 0.011 | | 0 |
|  | 138 | 0 | | 0.023 |
|  | 141 | 0 | | 0.023 |
|  | 144 | 0.021 | | 0 |
|  | 147 | 0.064 | | 0 |
|  | 150 | 0.053 | | 0 |
|  | 153 | 0.053 | | 0 |
|  | 156 | 0.255 | | 0.273 |
|  | 159 | 0.160 | | 0.205 |
|  | 162 | 0.138 | | 0.159 |
|  | 165 | 0.064 | | 0.068 |
|  | 168 | 0.053 | | 0.136 |
|  | 171 | 0.032 | | 0.045 |
|  | 174 | 0.021 | | 0 |
|  | 177 | 0.021 | | 0.023 |
|  | 180 | 0.032 | | 0 |
|  | 183 | 0.011 | | 0 |
|  | n | 94 | | 44 |
|  |  |  | |  |
| *TA60* | 69 | 0.011 | | 0 |
|  | 75 | 0.266 | | 0.257 |
|  | 78 | 0.117 | | 0.086 |
|  | 81 | 0.032 | | 0.114 |
|  | 84 | 0.287 | | 0.314 |
|  | 87 | 0.223 | | 0.2 |
|  | 90 | 0.032 | | 0 |
|  | 93 | 0.011 | | 0.029 |
|  | 96 | 0.021 | | 0 |
|  | n | 94 | | 35 |
|  |  |  | |  |
| *TA81* | 107 | 0.011 | | 0 |
|  | 110 | 0.011 | | 0.091 |
|  | 113 | 0.032 | | 0.023 |
|  | 116 | 0.191 | | 0.205 |
|  | 119 | 0.160 | | 0.273 |
|  | 122 | 0.298 | | 0.114 |
|  | 125 | 0.117 | | 0.045 |
|  | 128 | 0.032 | | 0.091 |
|  | 131 | 0.032 | | 0 |
|  | 137 | 0.011 | | 0 |
|  | 143 | 0.106 | | 0.023 |
|  | 152 | 0 | | 0.136 |
|  | n | 94 | | 44 |
|  |  |  | |  |
| *TA109* | 148 | 0.011 | | 0 |
|  | 154 | 0.011 | | 0 |
|  | 160 | 0.128 | | 0.122 |
|  | 163 | 0.266 | | 0.22 |
|  | 166 | 0.106 | | 0.049 |
|  | 169 | 0.011 | | 0 |
|  | 172 | 0.043 | | 0.098 |
|  | 175 | 0.106 | | 0.268 |
|  | 178 | 0.170 | | 0.195 |
|  | 184 | 0.032 | | 0 |
|  | 187 | 0.043 | | 0 |
|  | 190 | 0.011 | | 0 |
|  | 199 | 0.043 | | 0 |
|  | 202 | 0.021 | | 0.049 |
|  | n | 94 | | 41 |
|  |  |  | |  |
| *TA42* | 183 | 0.011 | | 0 |
|  | 186 | 0.777 | | 0.793 |
|  | 189 | 0.011 | | 0 |
|  | 201 | 0.128 | | 0.207 |
|  | 204 | 0.011 | | 0 |
|  | 216 | 0.021 | | 0 |
|  | 246 | 0.043 | | 0 |
|  |  |  | |  |
|  | n | 94 | | 29 |
|  |  |  | |  |
|  |  |  | |  |

* The previous survey refers to a separate sample of clinical isolates from the same area taken over one year previously (Mobegi *et al.* 2012 *Malaria Journal* 11:223).
